# Supplementary figures and images for: Genome compartmentalization predates species divergence in the plant pathogen genus Zymoseptoria
Source: BMC Genomics. 2020 Aug 26;21:588. doi: 10.1186/s12864-020-06871-w (PMC7448473; doi:10.1186/s12864-020-06871-w)

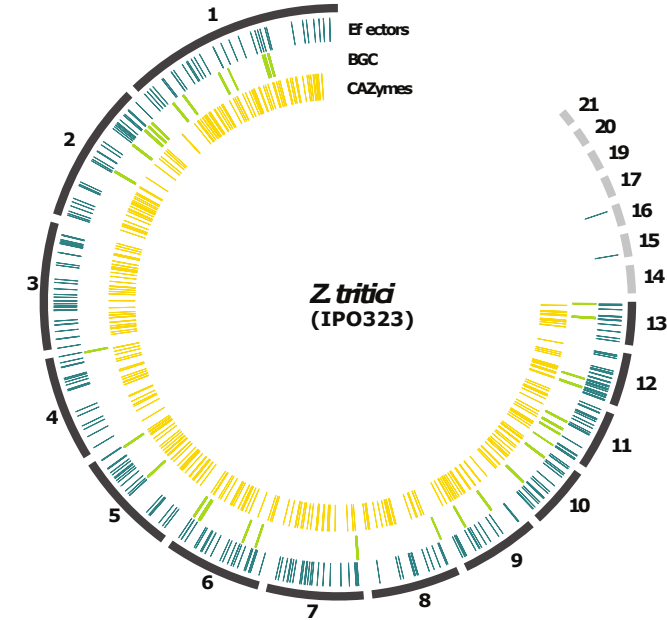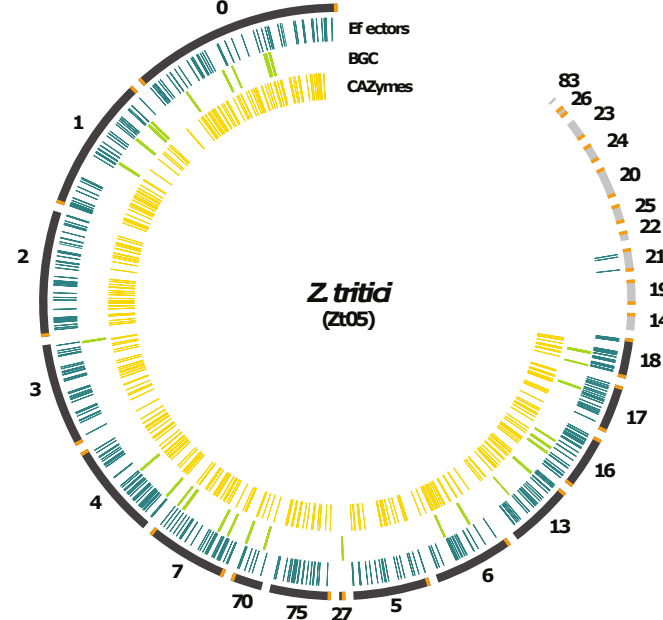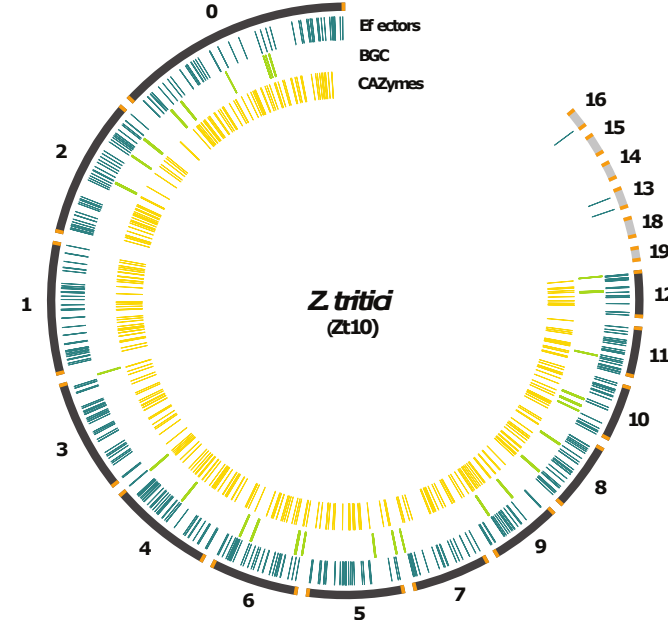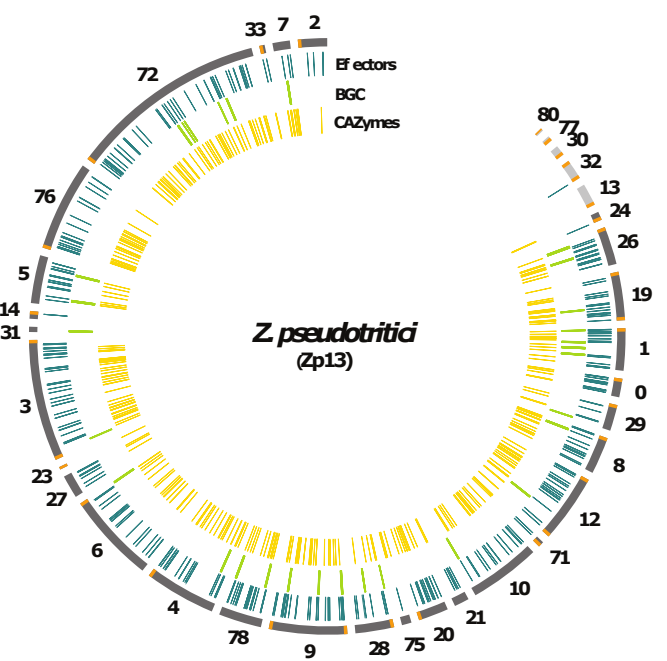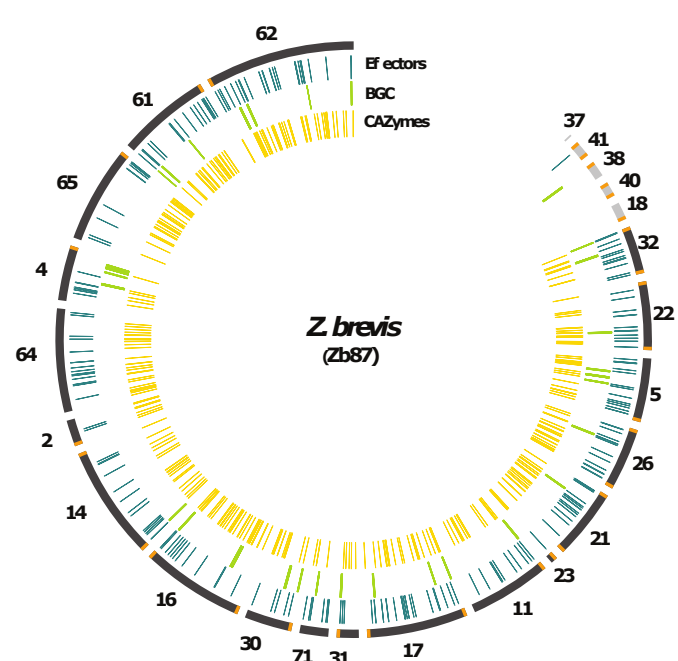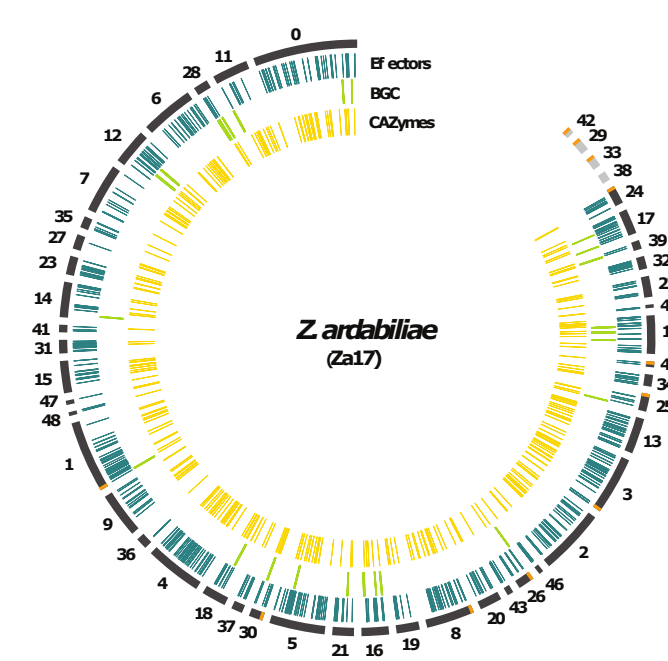

Supplement: Supplementary file 6 — Additional file 6: Figure S1. Plant-associated genes compartmentalization along the chromosomes. The first track represents core (dark grey) and accessory (light grey) chromosomes/contigs. Telomeric repeats are indicated in orange. Circles from outside to inside represent the position of: predicted effector genes (blue), biosynthetic gene clusters (BGC, green) and CAZymes (yellow) per 100 kb windows, respectively. [file 12864_2020_6871_MOESM6_ESM.pdf]

A

***Z. tritici***  
(IPO323)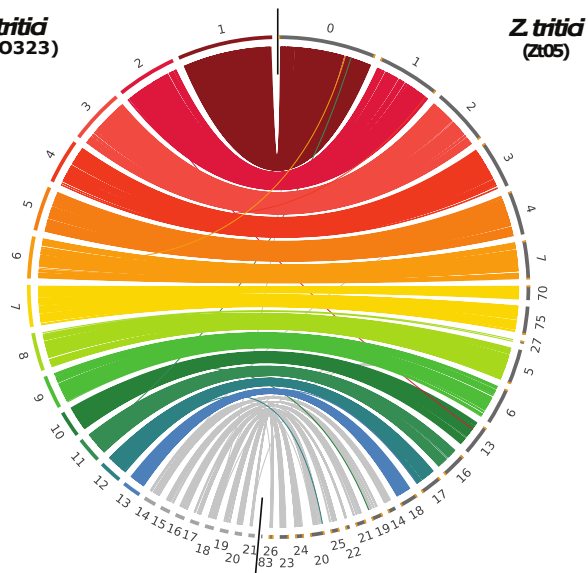

B

***Z. tritici***  
(IPO323)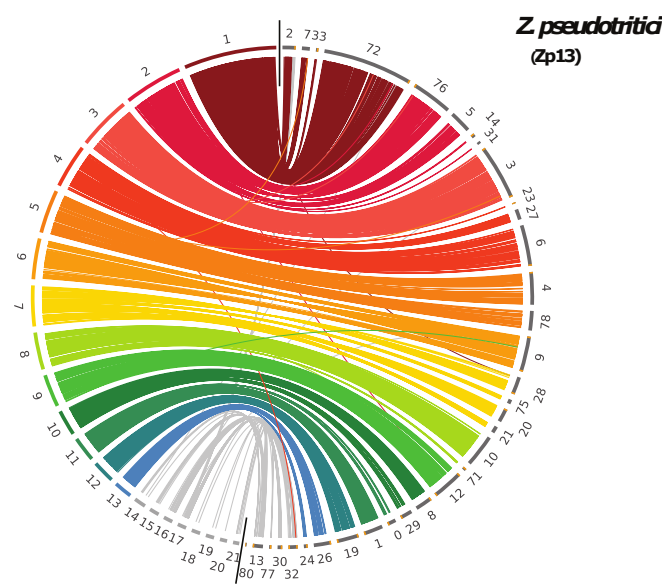***Z. pseudotritici***  
(Zp13)

C

***Z. tritici***  
(IPO323)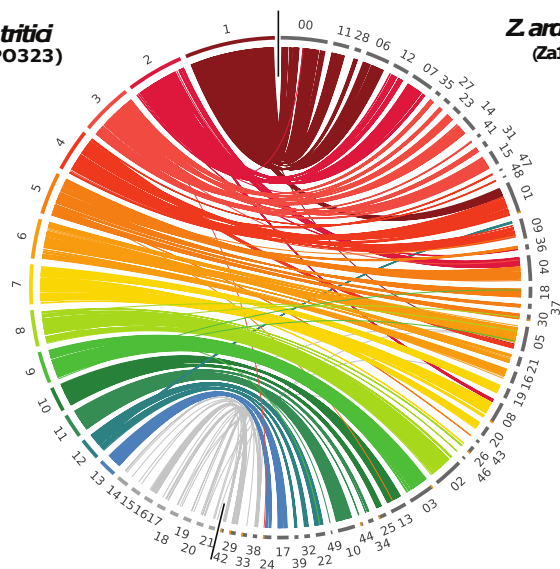***Z. ardabiliae***  
(Za17)

Supplement: Supplementary file 7 — Additional file 7: Figure S2. A) Intra-species synteny between the reference genome of Z. tritici and the genome of the Z. tritici isolate Zt05. Each color represents a different chromosome as based on the reference Z. tritici genome, except for accessory chromosomes, which are in grey. The connecting lines represent orthologs between each genome. The track between the chromosomes and connecting lines are predicted effector genes. Telomeric repeats are indicated in orange. B) Inter-species synteny between reference genome of Z. tritici and Z. pseudotritici. The arrows represent the large-scale inversions identified between the genomes of these two species. [file 12864_2020_6871_MOESM7_ESM.pdf]

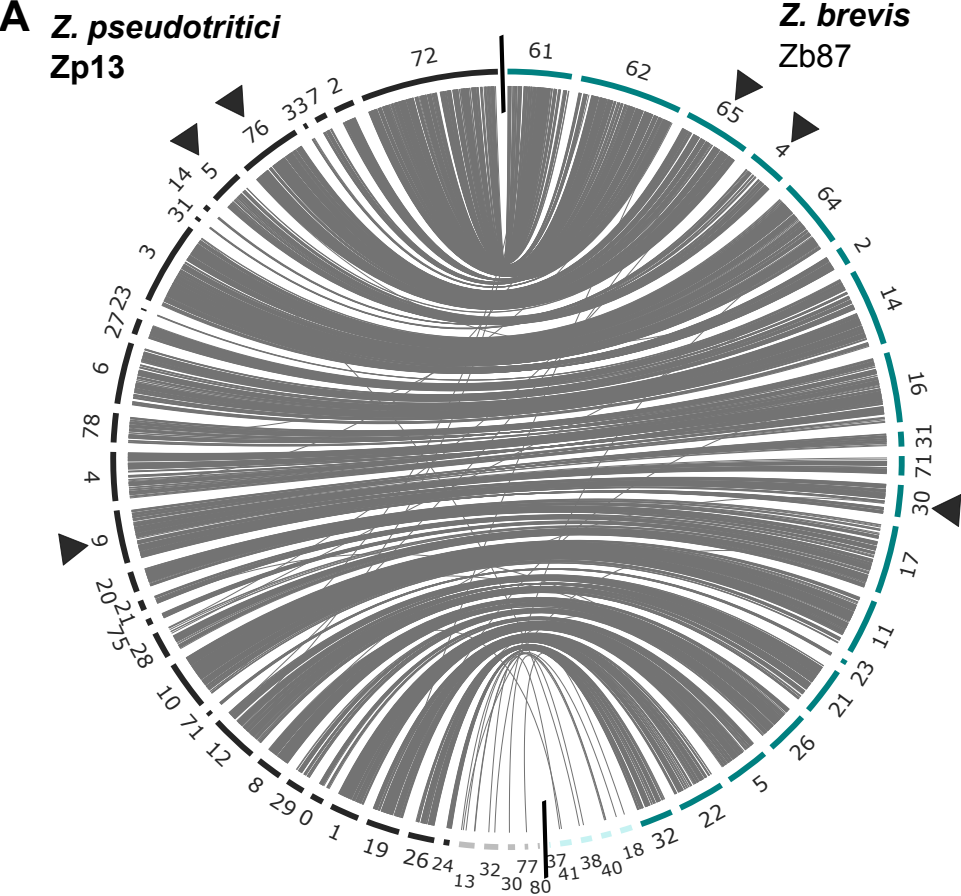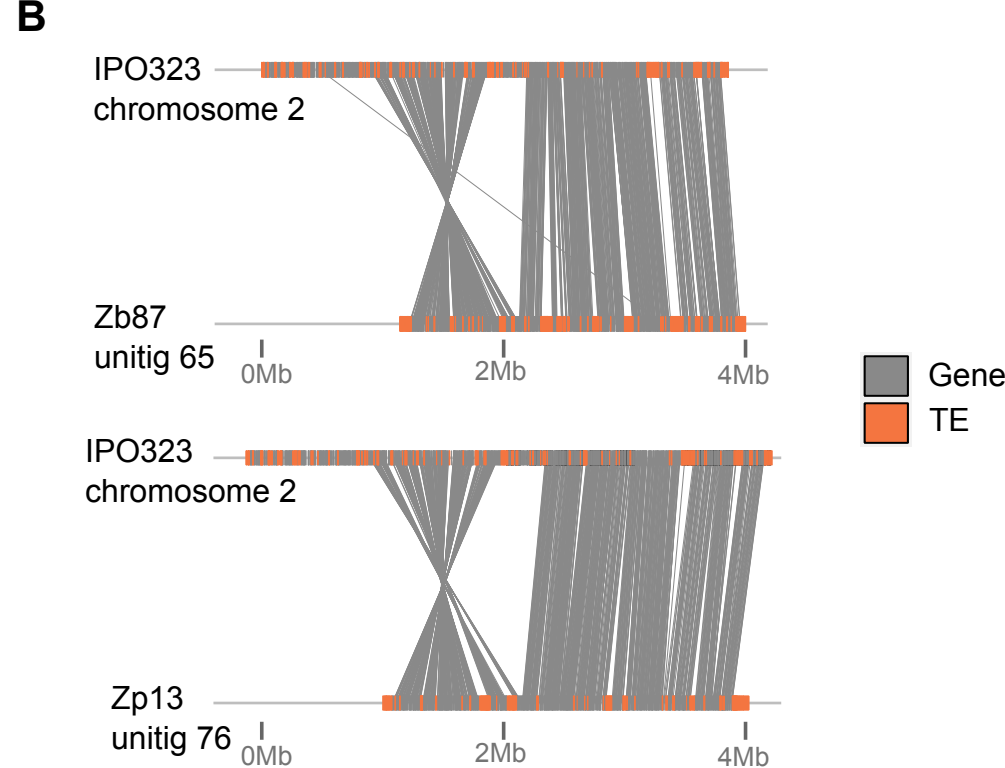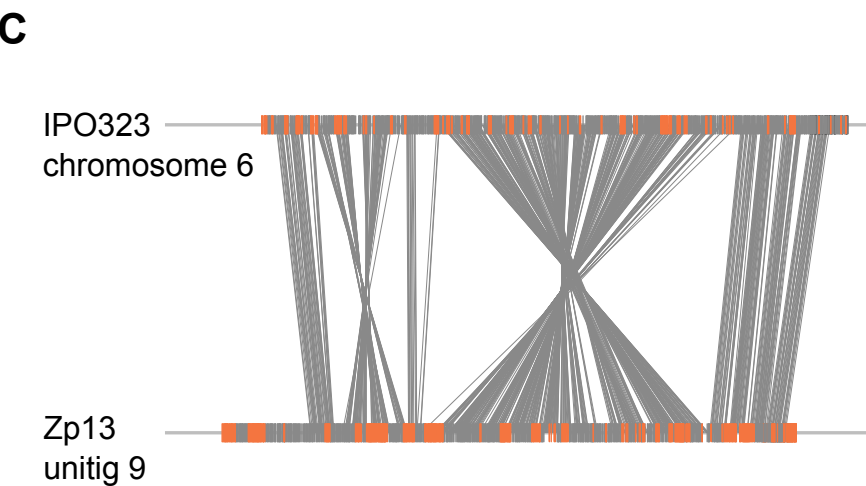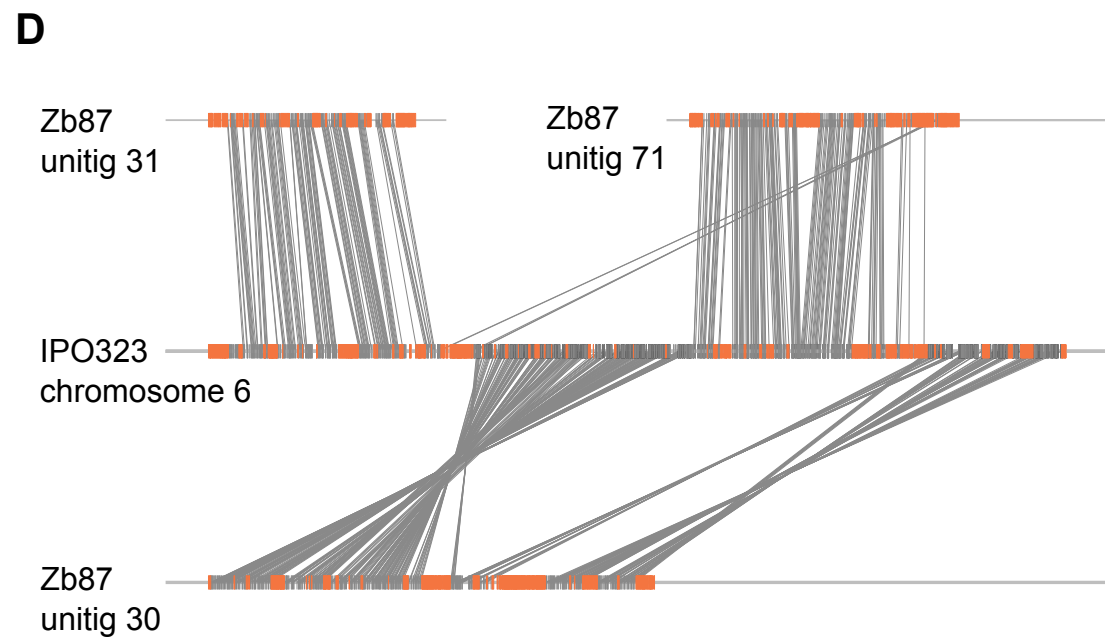

Supplement: Supplementary file 8 — Additional file 8: Figure S3. Inter-species synteny between genomes of Z. pseudotritici and Z. brevis. A) High level of nter-species synteny at the whole genome level between Z. pseudotritici (dark grey) and Z. brevis (blue). The arrows indicate the contigs identified as carrying rearrangement with Z. tritici but not between Z. pseudotritici and Z.tritici. B – D) Zoom-in on the contigs highlighted with the arrow in part A, showing the details of the pair-wise synteny of both Z.pseudotritici (Zp13) and Z. brevis (Zb87) with the Z.tritici reference (IPO323) chromosomes. [file 12864_2020_6871_MOESM8_ESM.pdf]

A

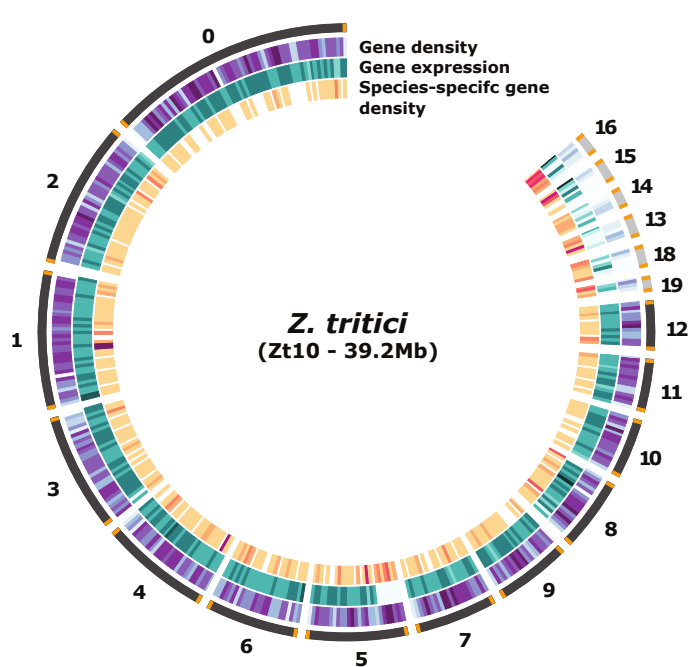

B

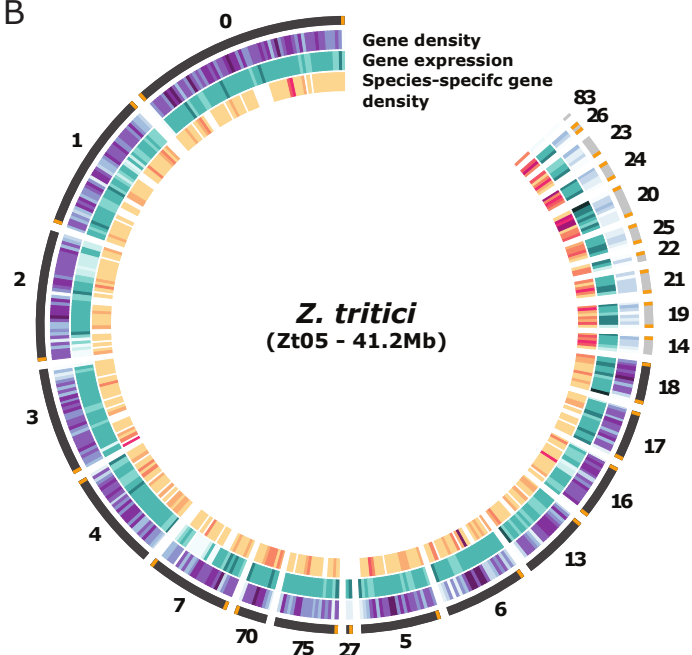

C

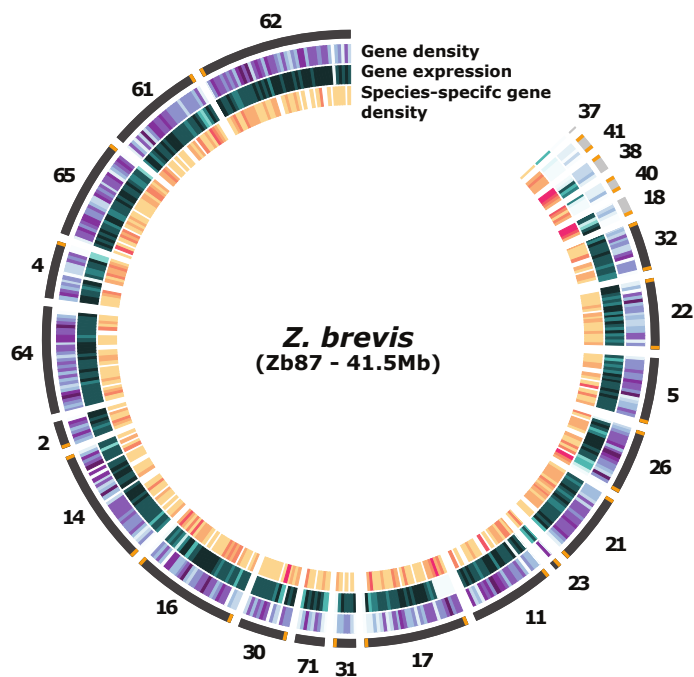

D

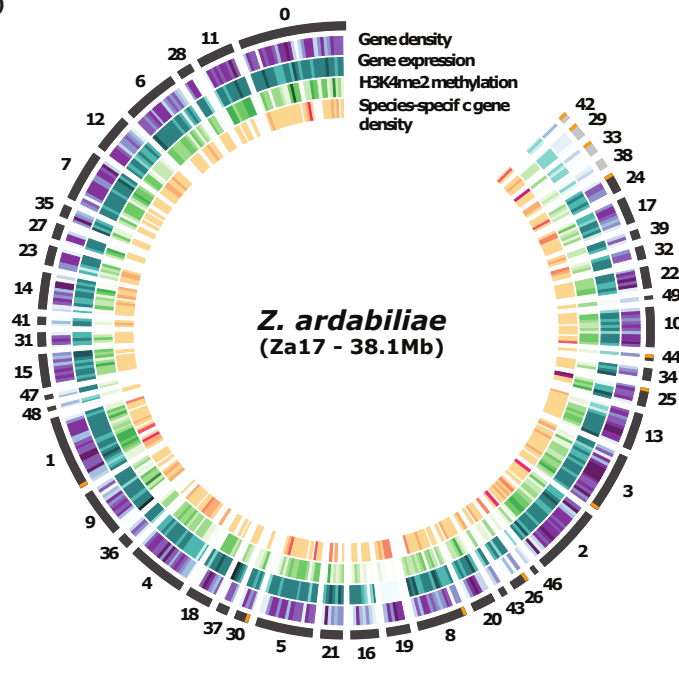

Supplement: Supplementary file 9 — Additional file 9: Figure S4. Genome architecture in A) Z. tritici Zt05, B) Z. tritici Zt10. C) Z. brevis Zb87 and D) Z. ardabiliae Za17. Circles from the outside to the inside represent respectively: gene density along chromosomes/contigs; gene expression in vitro (TPM); H3K4me2 distribution in vitro (only for Za17) and species-specific gene density per 100 kb windows. Contigs are ordered based on synteny with chromosomes of the reference strain IPO323. Telomeric repeats are indicated in orange. [file 12864_2020_6871_MOESM9_ESM.pdf]

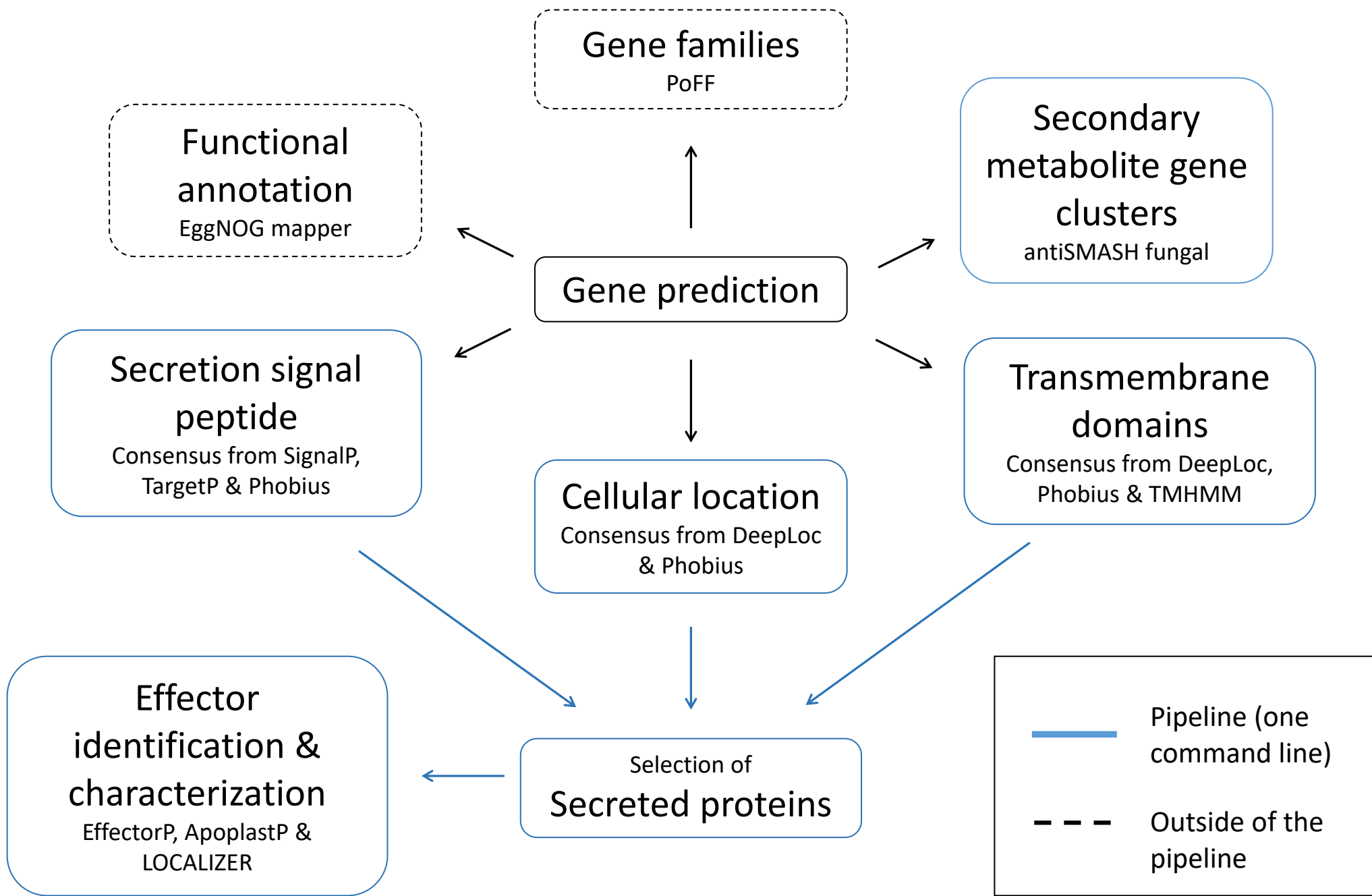

Supplement: Supplementary file 10 — Additional file 10: Figure S5. Simplified diagram of the pipeline used to predict the functions and subcellular localization of gene model products. [file 12864_2020_6871_MOESM10_ESM.pdf]

**A**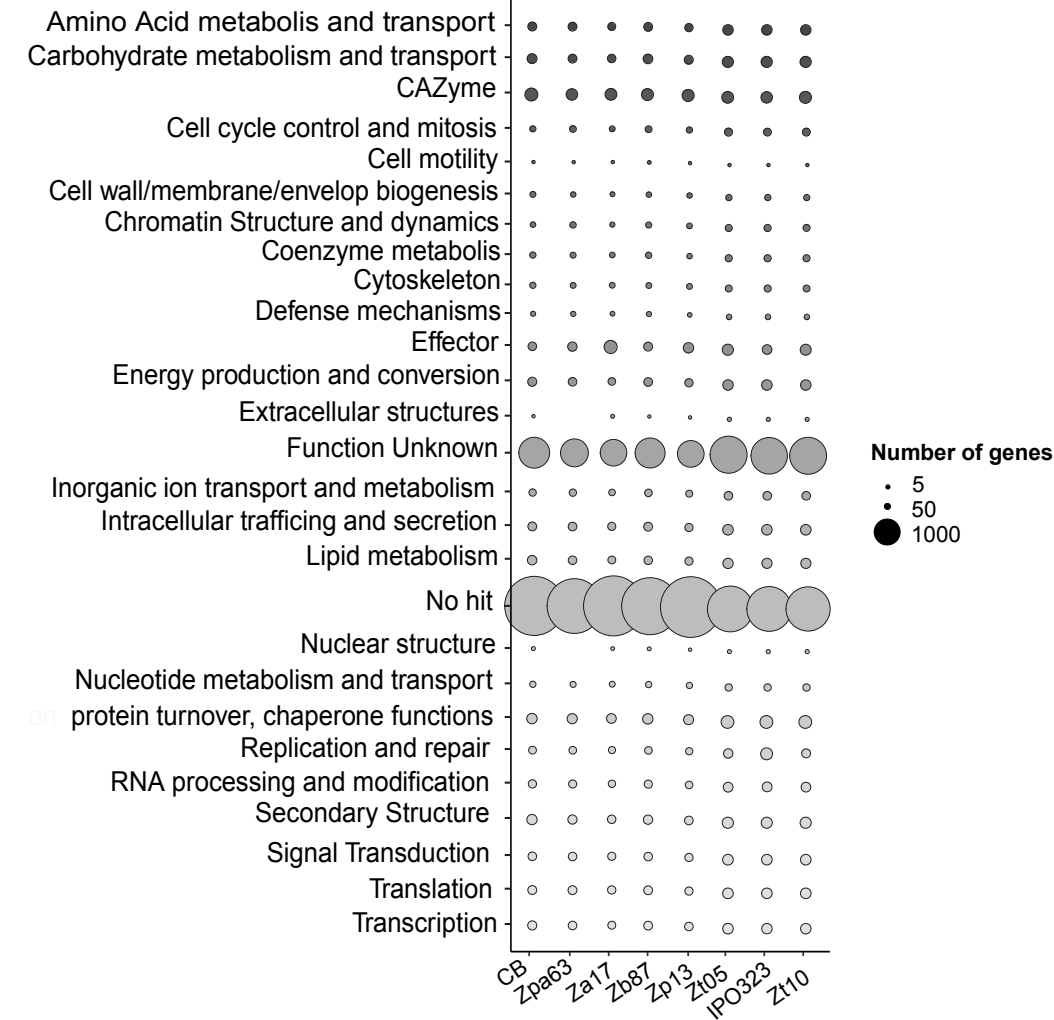**B**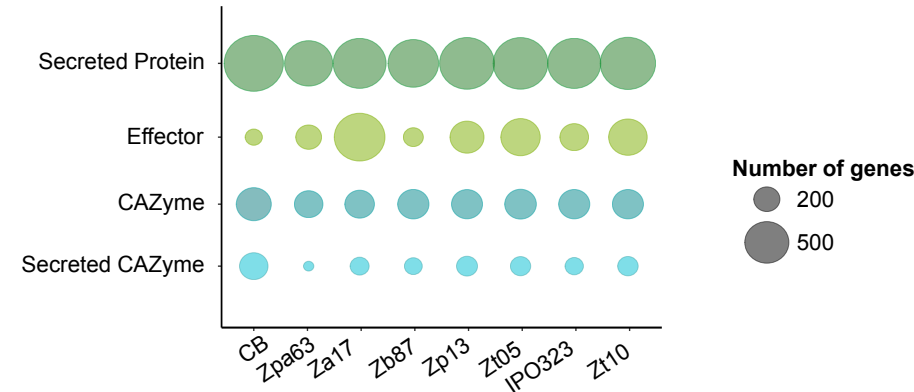**C**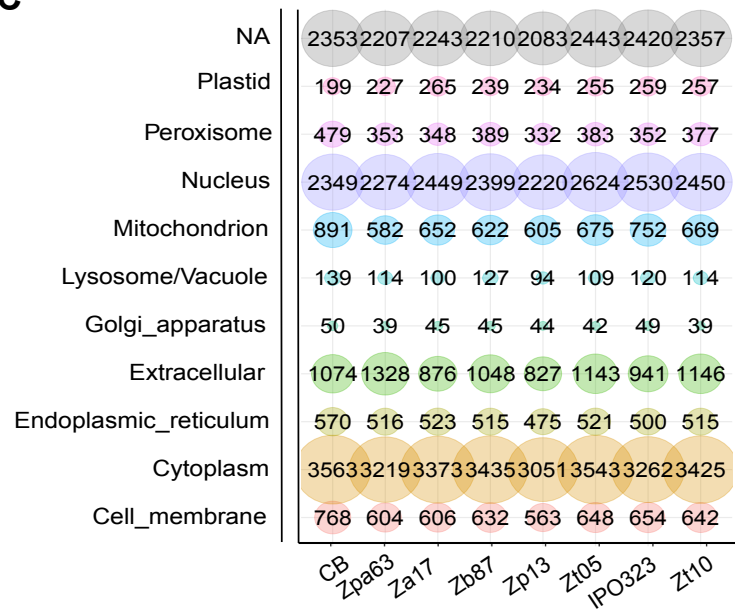

Supplement: Supplementary file 11 — Additional file 11: Figure S6. Functional gene categories in Zymoseptoria spp. genomes. A) The number of genes in Eggmapper COG categories in addition to Effectors and CAZymes. B) Pathogenicity-related genes of interest: secreted proteins, predicted effectors, secreted CAZymes and non-secreted CAZymes. C) Subcellular localization of predicted gene products. [file 12864_2020_6871_MOESM11_ESM.pdf]

## Biotrophic stages

## Necrotrophic stages

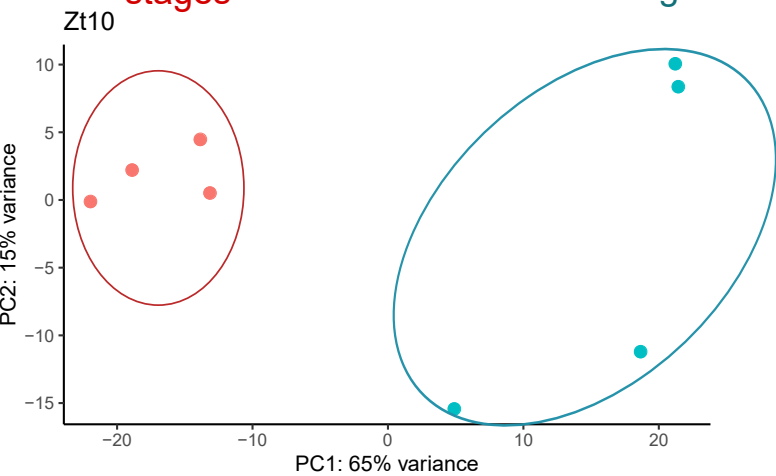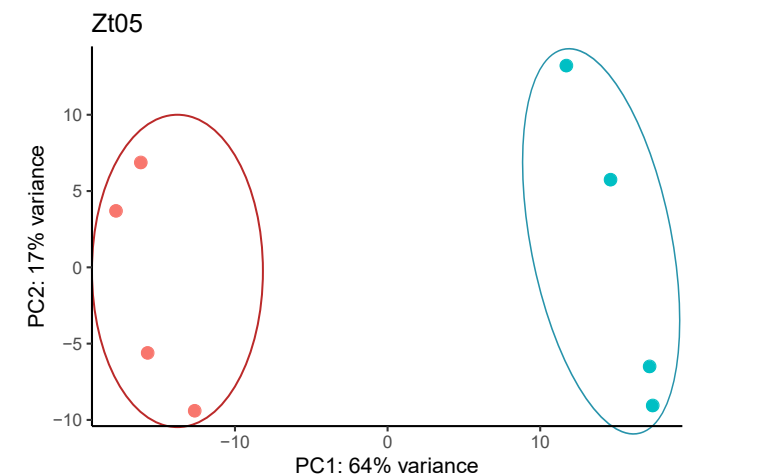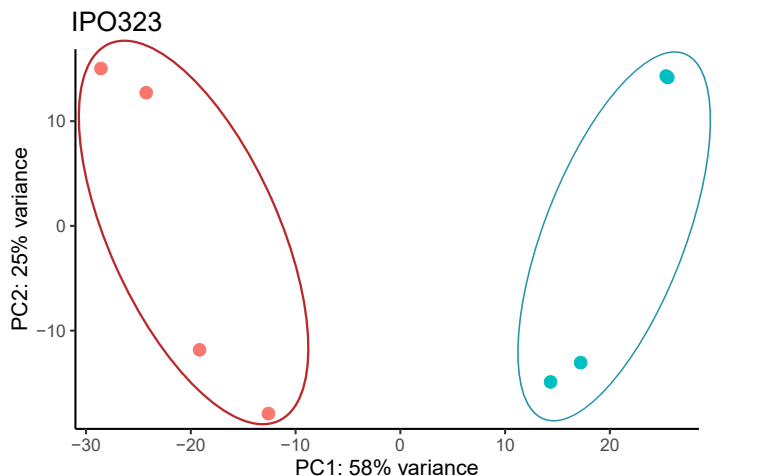

Supplement: Supplementary file 12 — Additional file 12: Figure S7. Principal component analysis of DESeq2 rlog transformed expression data. Principal component analysis (PCA) of Zymoseptoria tritici IPO323, Zt05 and Zt10 transcripts levels measured in biotrophic (red) and necrotrophic (blue) stages using RNA-sequencing. Reads detected per transcript (counts) were normalized using the size factor method used by DESeq2 package [73]. The PCA plot places biological replicates (four replicates per stage) along the two first PC axes explaining 58 to 65% of the variance (x-axis) and 15 to 25% (y-axis) of the variance within samples, respectively. [file 12864_2020_6871_MOESM12_ESM.pdf]
